# Assessment of Fetal Cell Chimerism in Transgenic Pig Lines Generated by *Sleeping Beauty* Transposition

**DOI:** 10.1371/journal.pone.0096673

**Published:** 2014-05-08

**Authors:** Wiebke Garrels, Stephanie Holler, Ulrike Taylor, Doris Herrmann, Heiner Niemann, Zoltan Ivics, Wilfried A. Kues

**Affiliations:** 1 Institut für Nutztiergenetik, Friedrich-Loeffler-Institut, Mariensee, Germany; 2 Paul-Ehrlich-Institute, Langen, Germany; University of Connecticut, United States of America

## Abstract

Human cells migrate between mother and fetus during pregnancy and persist in the respective host for long-term after birth. Fetal microchimerism occurs also in twins sharing a common placenta or chorion. Whether microchimerism occurs in multiparous mammals such as the domestic pig, where fetuses have separate placentas and chorions, is not well understood. Here, we assessed cell chimerism in litters of wild-type sows inseminated with semen of transposon transgenic boars. Segregation of three independent monomeric transposons ensured an excess of transgenic over non-transgenic offspring in every litter. Transgenic siblings (n = 35) showed robust ubiquitous expression of the reporter transposon encoding a fluorescent protein, and provided an unique resource to assess a potential cell trafficking to non-transgenic littermates (n = 7) or mothers (n = 4). Sensitive flow cytometry, fluorescence microscopy, and real-time PCR provided no evidence for microchimerism in porcine littermates, or piglets and their mothers in both blood and solid organs. These data indicate that the epitheliochorial structure of the porcine placenta effectively prevents cellular exchange during gestation.

## Introduction

Microchimerism refers to the presence of a small ratio of foreign cells (<1∶100) within the tissues of a host organism. Microchimerism can occur after iatrogenic interventions, such as transplantation or transfusion, or naturally between twins, or mother and fetus. In utero, cell transfer between littermates or materno-fetal cell exchange can have important consequences for the function of the immune system, health of the offspring and for tissue compatibility [1]–[3]. Microchimerism is found in women [4], [5], and in their progeny by genotyping and detection of transferred tumor cells [6], [7]. The human hemochorial placenta, in which maternal blood is in direct contact with the chorionic trophoblast, facilitates trafficking of cells and/or macromolecules, such as antibodies [7], [8].

Most of the domestic species possess an epitheliochorial placenta [9], which consists of maternal and fetal epithelia and is thought to be much tighter than the hemochorial placenta. Nevertheless, cell trafficking has been described in these species [10]. In cattle, fetal microchimerism was observed in monochorionic twins [11]–[13]. Fetal microchimerism has also been reported in goats [14]. However, cell transfer in multiparous, multichorionic species like the domestic pig has only rarely been studied. A recent report suggested microchimerism in porcine littermates under specific experimental situations [15], in which xenogenic human cord blood derived cells had been injected into the peritoneum of porcine fetuses around day 40 of gestation. Evidence for long-term maintenance of human cells in the treated animals, but also in untreated littermates was reported [15].

The domestic pig is an important model for biomedical studies and preclinical cell therapy approaches [16]–[19]. Fetal chimerism could be biologically relevant in the pig, specifically for immunology, since the consequences of fetal microchimerism might affect immunity, immune surveillance and tissue repair [8], [20]–[26]. In recipient cows, which were pregnant with transgenic embryos, transgene-specific DNA sequences were found in blood of 50% of the surrogate mothers [27]. In another study, enhanced green fluorescent protein (EGFP) was detected in maternal parts of the placenta [28]. These findings suggest that transplacental leakage of fetal DNA and proteins into the maternal circulation can occur in cattle despite the epitheliochorial placenta. Whether the leakage of fetal DNA correlated with transfer of cells and long-term persistence of fetal cells is not yet known.

Here, we assessed potential inter-fetal and feto-maternal cell transfer in lines of transgenic pigs expressing a Venus fluorophore reporter [29]. Venus is a derivative of the enhanced yellow fluorescent protein (EYFP), and is compatible with live imaging. The *Venus* construct was integrated into the porcine genome by *Sleeping Beauty* transposition and was found to be ubiquitously expressed [29]–[31]. Offspring from two founder boars, each carrying three monomeric reporter transposons in their genome were used for this study [29], [32]–[34]. The faithful expression of Venus in a ubiquitous manner is an excellent tool for the identification of even few transgenic cells in circulation and solid organs of non-transgenic littermates or wild-type animals.

## Materials and Methods

### Ethics Statement

Animals were maintained and handled according to German and international laws regulating animal welfare and genetically modified organisms (GMO), and all experiments were approved by an external animal welfare committee at the Niedersächsisches Landesamt für Verbraucherschutz und Lebensmittelsicherheit (LAVES) in Oldenburg, Germany (AZ 33.9-42502-04-09/1718).

### Collection of Ejaculated Sperm and Artificial Insemination

Sperm-rich fractions were collected from boars using a dummy and by gloved – hand technique [31], [32]. The semen samples were extended with Androhep (1∶1) and transported to the laboratory at 37°C. Sperm concentration was determined by NucleoCounter SP-100 system (ChemoMetec, Denmark). Inseminations of wild-type sows at oestrus were performed according to standard procedure.

### Fluorescence Microscopy and Macroscopic Excitation of Venus Fluorochrome

For fluorescence microscopy, images were obtained by an Olympus BX 60 (Olympus, Hamburg, Germany) fluorescence microscope equipped with a 12-bit digital camera (Olympus DP 71). For specific excitation of live *Venus*-transgenic piglets and pigs, a blue floodlight LED (40 W; eurolite Germany, Germany) and an electronic camera (Canon Powershot) equipped with a yellow emission filter was used.

### Identification of Venus Positive Cells by Flow Cytometry

Flow cytometry analysis of leukocytes was performed using a FACScan (BD Bioscience, Heidelberg, Germany) equipped with an argon laser (488 nm, 15 mW) [31], [32]. Samples were diluted to 0.5×10^6^ cells/ml and measured in triplicates acquiring 50 000–300 000 cells per sample. Membrane impaired cells were excluded from analysis by staining with propidium iodide (20 µM).

### Fluorescence Histology

Tissue samples (∼5 mm^3^) were snap frozen in liquid nitrogen and then fixed in 3.6% formaldehyde for 12–24 hours. After washing in phosphate-buffered saline, the samples were soaked in 30% sucrose for 24 hours, then frozen in tissue-tec (Hertenstein, Germany), and 10 µm sections were prepared in a cryostat (Micom Laborgeräte, Walldorf, Germany) and dried on glass slides. The sections were mounted with Vecta-shield (H-100) and viewed under a fluorescence microscope.

### Genotyping of Offspring

Genomic DNA was isolated with the proteinase K method, digested with NcoI, separated on a 0.6% agarose gel by electrophoresis and blotted on a polyvinylidene fluoride (PVDF) membrane. Then a transgene specific probe labelled with digoxigenin was used for hybridization as described [29], [31], [32].

### Real Time-PCR

Genomic DNA was isolated from leukocytes with the proteinase K method. The PCR mix in each well included 15 µl of 2x Power SYBR Green PCR Master Mix (Applied Biosystems), 11.4 µl dH_2_O, 1.6 µl each of the primer pairs (5 µM), 2 µl of DNA (25 ng) in a total volume of 30 µl. Primer and PCR characteristics are summarized in Table S1. The PCR program included denaturation and activation of the Taq polymerase for 10 min at 95°C followed by 45 cycles of 95°C for 15 s and the appropriate annealing temperature given in Table S1 for 1 min. Then a dissociation curve of the product was assessed (ABI 7500 Fast Real-Time System, Applied Biosystems).

## Results

**Figure 1:**
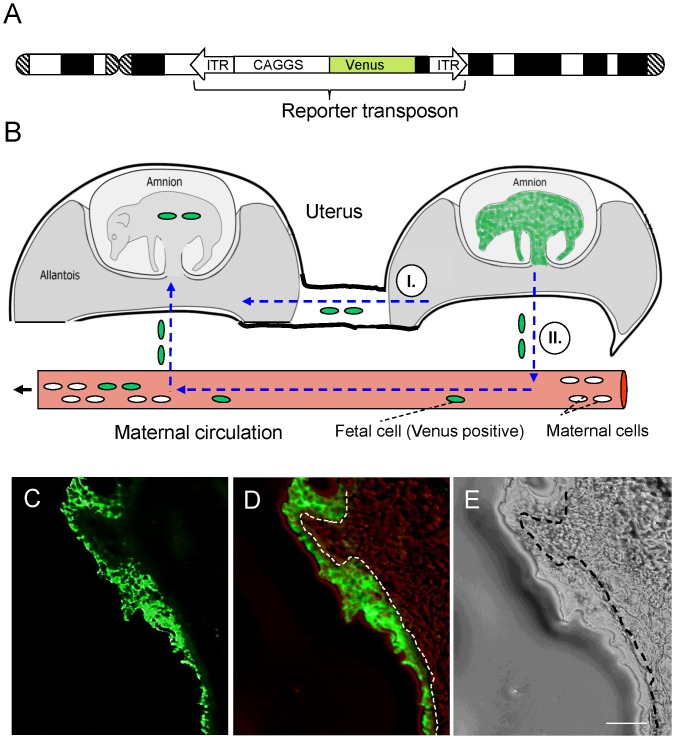
Reporter transposon and potential routes of inter-fetal and feto-maternal cell trafficking in the pig. A) Schematic depiction of a monomeric reporter transposon in chromosomal context. The SB transposon consists of *CAGGS* promoter, *Venus* cDNA and polyadenylation sequence flanked by SB inverted terminal repeats (ITRs). Drawing not at scale. B) Fetal cells might traffic directly between fetuses (I.) or via the maternal circulation (II.). In both cases the non-transgenic fetuses should carry Venus-expressing cells. In case (II.), the mother should also show cell chimerism. For simplification only two fetuses are depicted and only the transgenic fetus is shown in green, however, cells of amnion and allantois are also Venus positive. C) Specific Venus fluorescence in embryonic allantois at day 25 of gestation. Cryosection of a *CAGGS-Venus* transgenic implantation in a wild-type sow. D) Overlay of fluorescence and brightfield views. E) Corresponding brightfield view. A dotted line indicates the border between embryonic and maternal tissue. Bar = 10 µm.

Two transgenic founders, each carrying three monomeric integrations of the *cytomegalovirus enhancer, chicken beta actin* hybrid promoter (*CAGGS)-Venus* transposon, were used for collection of semen, and six wild-type sows were artificially inseminated. This resulted in 5 pregnancies that went to term and yielded a total of 44 piglets, of which 35 were transgenic and 9 were non-transgenic. Seven of the non-transgenic littermates were analysed in detail for signs of fetal chimerism (Tab. 1). An additional pregnancy of a wild-type sow carrying *GAGGS-Venus* transgenic embryos was interrupted at day 25 of gestation. Out of 12 fetuses, 10 were transgenic and expressed the Venus reporter in fetal tissue, amnion and allantois (Fig. 1). The ratio of transgenic to non-transgenic genotypes fitted with the independent inheritance of three monomeric transposon copies according to Mendelian rules as shown recently [32].

**Table 1:** Assessment of potential chimerism in non-transgenic littermates and sows.

Animal ID	Status	FACS (Venus pos. leukocytes)	Tissue sections (Venus pos. cells)	Real time PCR (Ct)
#506	Non-tg littermate	<1∶ 200 000	<1∶ 50 000	40.1±1.1
#512	Non-tg littermate	<1∶ 240 000	<1∶ 50 000	∞
#522	Non-tg littermate	<1∶ 180 000	<1∶ 50 000	36.2±0.9
#524	Non-tg littermate	<1∶ 200 000	<1∶ 50 000	38.5±1.0
#527	Non-tg littermate	<1∶ 140 000	<1∶ 50 000	37.2±1.9
#531	Non-tg littermate	<1∶ 120 000	<1∶ 50 000	38.0±0.4
#533	Non-tg littermate	<1∶ 100 000	<1∶ 50 000	36.2±0.9
#515	Tg littermate	All (99.97%)	All	<25
#517	Tg littermate	All (99.90%)	All	<25
Sow #404§	Mother of #506, #531, #533	<1∶ 300 000	<1∶ 50 000	41.6±0.9
Sow #408	Mother of #512, #515, #517	<1∶ 120 000	<1∶ 50 000	40.1±1.8
Sow #412	Mother of #522, #524	<1∶ 50 000	<1∶ 50 000	38.4±1.2
Sow #420	Mother of #527	n.d.	n.d.	∞
Sow 1	Unrelated control	<1∶ 200 000	<1∶ 50 000	38.3±1.9
Sow 2	Unrelated control	<1∶ 200 000	n.d.	∞
Sow 3	Unrelated control	<1∶ 200 000	n.d.	n.d.

ID, unique identification number; Ct, threshhold cycle in a quantitative real time PCR;

∞, no threshold cycle reached during PCR;

n.d., not done;

§ , sow with two “transgenic” pregnancies.

In principle, either direct cell trafficking between fetuses or indirectly via circulation of the mother is conceivable (Fig. 1). First, the robust and ubiquitous expression of Venus reporter in transgenic offspring was confirmed. All transgenic animals showed homogenous expression of the Venus protein, which could be detected already by direct fluorescence of pigs in the barn, but also by FACS measurements, cryosections, and with molecular methods [32], [33]. Terminally differentiated gametes of these animals were also Venus positive [32]. A macroscopic inspection of inner organs revealed tissue-specific expression levels of Venus [32].

The 7 vital non-transgenic offspring (Tab. 1) were of particular interest for the assessment of fetal chimerism. Since all non-transgenic offspring were obtained from litters with a surplus of transgenic littermates, potential microchimerism should be reflected by a certain ratio of transgenic cells in the non-transgenic animals and/or in their wild-type mothers (Fig. 1).

To investigate the potential scenarios for cell chimerism, blood samples were collected from pregnant sows at two different time points of gestation (4 weeks and 12 weeks) and after they gave birth, as well as from the non-transgenic piglets (Tab. 1), and were analysed by flow cytometry, PCR and fluorescence microscopy. In parallel, blood and tissue samples from two transgenic animals were analysed. The samples from transgenic animals were processed separately to prevent any cross-contamination during handling.

**Figure 2:**
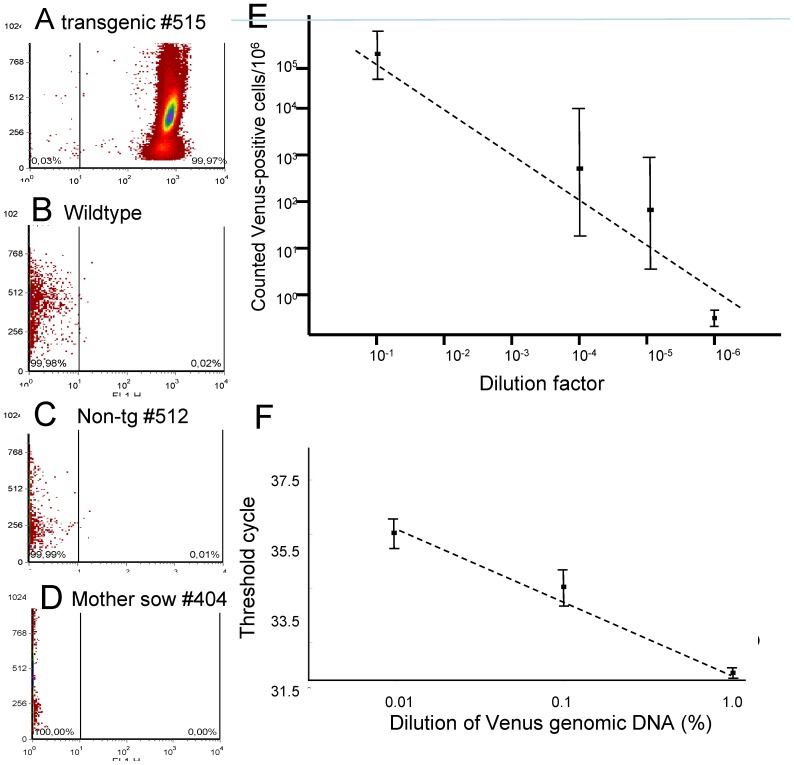
Flow cytometric measurement of porcine leucocytes for Venus-positive cells. A) Venus transposon transgenic pig (#515), B) Wild-type pig, C) Non-transgenic littermate, D) Sow #404 (delivered two litters of transposon piglets), E) Determination of the detection limit of flow cytometry. Leukocytes from a wild-type animal were spiked with decreasing amounts of Venus-positive leukocytes (n = 3 technical and biological replicates for each dilution). The dotted line indicates the theoretically expected cell counts. A detection limit of 1 Venus cell in 100 000 wild type cells was determined. F) RT-PCR of a dilution series of *Venus* gDNA in wildtype DNA.

First, we determined the expression of Venus in leukocytes of transgenic and non-transgenic littermates. In leukocytes isolated from transgenic animals, all cells exhibited prominent Venus fluorescence which was about two log orders higher than in wild-type cells (Fig. 2). In artificially spiked leukocyte preparations (transgenic : non-transgenic cells = 1∶10^1^ to 1∶10^6^) the Venus-positive cells could unequivocally be identified with a detection limit of 1 Venus-positive cell per 10^5^ wild-type leukocytes (Fig. 2). Flow cytometric results were further substantiated by re-analysis via fluorescence microscopy (Fig. S1). In leukocytes from non-transgenic animals (n = 7) Venus-positive cells were never found, even by analysing 50 000–300 000 cells per measurement (2–3 replicates per animal).

In addition, leukocytes from three mothers (animal #404 had been pregnant twice and delivered two transgenic litters) and three unrelated wild-type sows (negative controls) were analysed by flow cytometry. To scan for rare events, a minimum of 50 000 cells and a maximum of 300 000 cells were counted. The negative control samples, but also the samples from the mothers did not reveal any Venus-positive blood cells (Fig. 2). Even a sow (#404), which had delivered two transgenic litters did not show any Venus positive leukocytes.

To assess the possibility that non-transgenic littermates carry transferred transgenic cells, which do not express the transgene, and consequently would not be detected by flow cytometry, genomic DNA (gDNA) was isolated and analysed by sensitive real time PCR. The highest amount of gDNA per reaction was 25 nanograms (ng), higher amounts of gDNA decreased amplification efficiency. As positive control, *polyadenylate polymerase* (*PAPOLA*) was amplified from all samples. The detection limit of transgenic sequences was determined by assaying a serial dilution of transgenic DNA in wild-type gDNA. It was calculated that the detection limit was 1 transgenic sequence in 10 000 copies of wild-type gDNA (Fig. 2F), corresponding to a threshold cycle of 35.5. Blood was collected from all non-transgenic littermates and wild-type mothers twice at 4 weeks intervals and was used to isolate gDNA. RT-PCR reactions were run in duplicates or triplicates. However, no indication for the presence of *Venus*-specific DNA was found either in samples from non-transgenic littermates or from the sows; all samples produced threshold cycles above 36.

**Figure 3:**
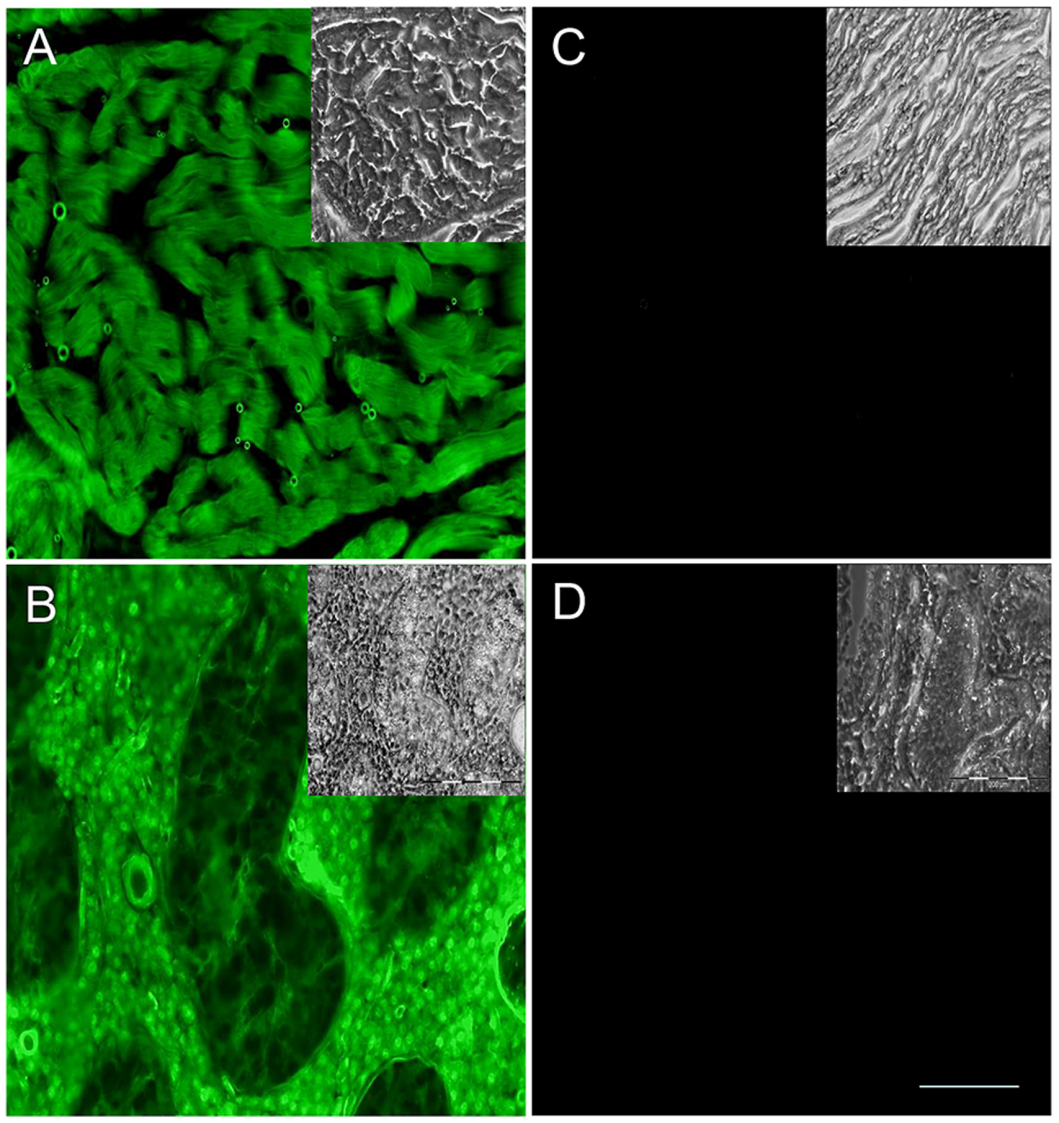
Assessment of reporter-positive cells in solid organs. A, B) Cryosections of heart and testis of a Venus-transposon pig are depicted under specific excitation of Venus and brightfield conditions (insets). C) Heart, and D) testis sections of a non-transgenic littermate were recorded under identical camera settings. White bar = 50 µm.

To assess the possibility that solid organs host transferred cells, the seven non-transgenic littermates and three carrier sows were sacrificed and heart, liver, kidney, brain, lung, spleen, skin, muscle and gonade were analysed. In parallel, organ samples from two transgenic piglets were processed. For microscopic analysis, tissues sections (10 µm) were prepared and 20 sections per organ were analysed for Venus-positive cells. Each section covered a minimum of 50 000 cells, thus an estimated number of 1 000 000 cells were screened per organ. Venus-expressing cells were not found in the non-transgenic littermates (Fig. 3).

## Discussion

The present findings provide no evidence of fetal cell trafficking in the pig. Depending on the applied technique different detection limits were determined. Real time PCR had a detection limit of 1 target sequence per ∼10 000 genome copies, assuming that one diploid cell contains 6 picogram of DNA. The histological examination was estimated to have a detection limit of 1∶50 000 cells, and for the flow cytometry a detection limit of 1 in 100 000 cells was determined. The prerequisite for the histological and flow cytometric measurements was the robust and ubiquitous Venus expression in somatic, and germ line cells of the *Venus* transgenic pigs [29], [32]–[34]. The prominent Venus expression allowed the unambiguous detection and identification of transgenic cells.

Whereas flow cytometric and fluorescence microscopic detection depend on the expression of the transgene, real time PCR allows detection of non-expressing cells or free DNA. In contrast to data in cattle, where Y chromosome-specific DNA was detected in up to 73% of blood samples from naturally mated heifers carrying conventional bull calves, and a transgene-specific sequence was detected in up to 50% of recipient cows carrying transgenic fetuses [27], the present study revealed no foreign DNA or cells in either the carrier sows or the non-transgenic piglets. Potentially, the different morphological structures of the porcine placenta diffusa and the bovine placenta cotyledonaria contribute to these divergent results. The premature rupture of fetal membranes and cell exchange shortly before birth might depend on the placenta form [35]. However, whether or not fetal cells exist over long periods of time in bovine foster mothers was not yet investigated [27], [28].

Similarly to the present results, no fetal chimerism could be detected between transgenic porcine littermates [36], and in caprine surrogate mothers [37].

In contrast to the absence of fetal microchimerism in the present study, McConico et al. [15] reported fetal chimerism in the pig after xenogenic transplantation. In untreated littermates a frequency of xenogenic cells of 1∶10 000–1∶100 000 was reported [15]. In this setting, human cord blood cells were injected into the peritoneum of porcine fetuses by surgical intervention around day 40 of gestation. Potentially, the iatrogenic intervention [15], [38] itself may have facilitated distribution of human cells to untreated littermates. Consequently, in iatrogenic interventions the carryover of cells to untreated littermates and mothers should be considered.

Previously, cell tracking experiments with genetically marked cells have been performed in mice to assess fetal chimerism [39]–[42]. After mating of *CAGGS-EGFP* transgenic males with wild-type females, the highest ratio of fetal cells was 1% in leukocytes prepared from maternal blood during pregnancy and 0.3% in leukocytes after birth [41]. In solid organs of pregnant females Sunami et al. [42] determined 4–191 fetal cells per 100 000 maternal cells, whereas Fujiki et al. [40] found ∼1 fetal cell per 1 million maternal cells in leukocytes and 10–40 fetal cells per 1 million maternal cells from lung, liver and spleen at day 18 of gestation. It was concluded that the availability of transgenic lines with robust EGFP expression in combination with flow cytometric analysis is the most versatile detection method [43]. Most likely, the frequent fetal chimerism in mice is caused by the murine hemichorial structure of the placenta. In species, where the transgenic technology for genetic labelling is not available, PCR approaches for the detection of Y chromosome-specific sequences, or of informative polymorphic sequences are the gold standard for the assessment of fetal chimerism [44], [45].

During normal prenatal development the epitheliochorial placenta of the pig seems to effectively prevent cell trafficking between fetuses and mother. Thus the results of the present study substantiate the hypothesis that the epitheliochorial placenta is non-permeable for fetal cells. In addition, no signs for inter-fetal cell transfer were found.

## Supporting Information

Figure S1 **Microscopic detection of Venus-positive leukocytes in spiking experiments.** A) Leukocytes of animal #517 (transgenic littermate) shown under specific fluorescence excitation of Venus, and B) under brightfield illumination. Note that all leukocytes are Venus-positive. A remaining erythrocyte (asterix) expressed no fluorescence [32]. C) Leukocytes of animal #408 (wildtype sow) spiked with leukocytes from #517 (ratio #408 : #517 = 100∶ 1). A Venus-positive leukocyte (+) is indicated. D) Corresponding brightfield illumination of C). Bar = 25 µm.
(TIF)

Table S1 **Primer pairs used for RT-PCR.**
(DOCX)
