# Supplementary figures and images for: Assessment of Fetal Cell Chimerism in Transgenic Pig Lines Generated by Sleeping Beauty Transposition
Source: PLoS One. 2014 May 8;9(5):e96673. doi: 10.1371/journal.pone.0096673 (PMC4014516; doi:10.1371/journal.pone.0096673)

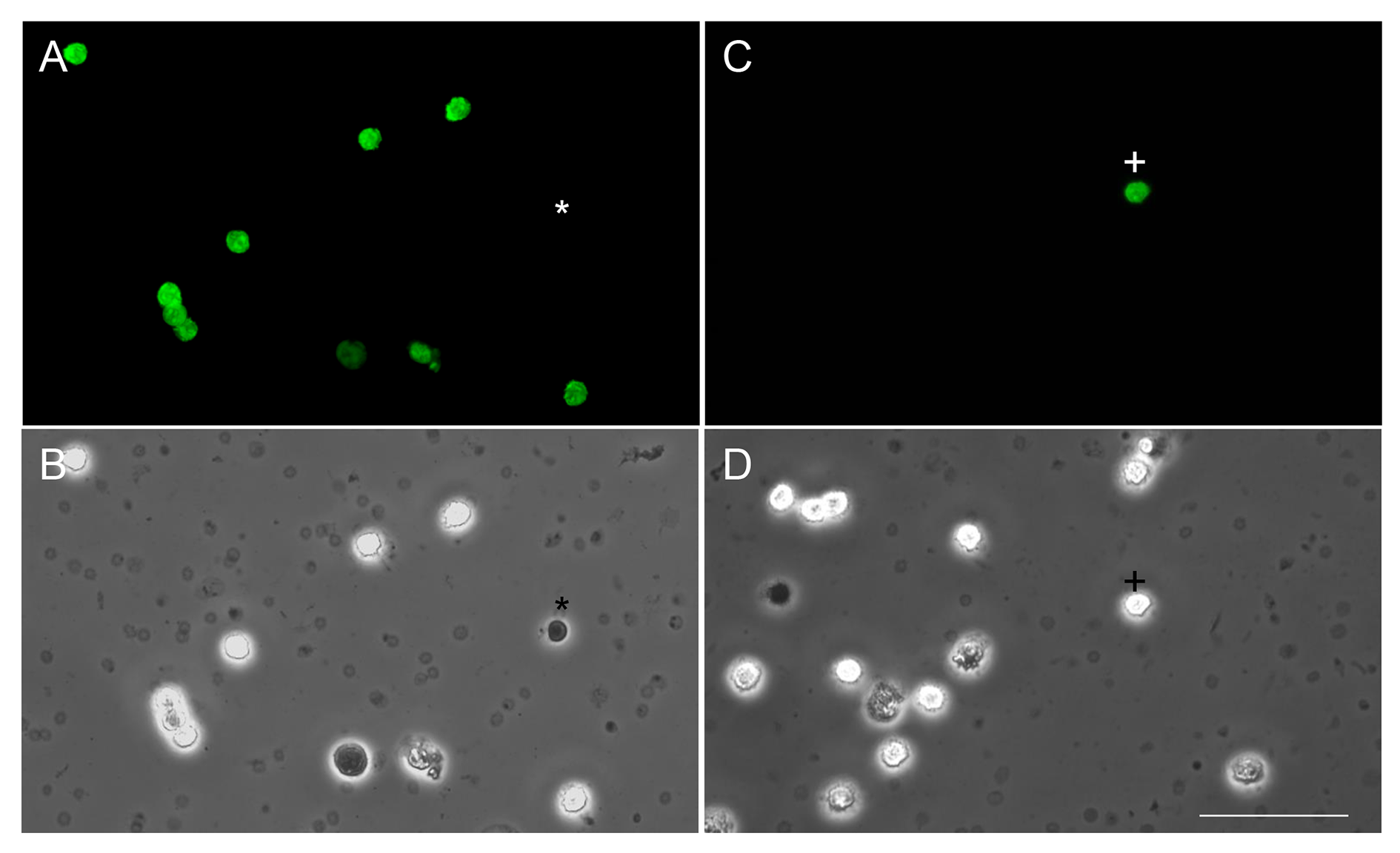

Supplement: Figure S1 — Microscopic detection of Venus-positive leukocytes in spiking experiments. A) Leukocytes of animal #517 (transgenic littermate) shown under specific fluorescence excitation of Venus, and B) under brightfield illumination. Note that all leukocytes are Venus-positive. A remaining erythrocyte (asterix) expressed no fluorescence [32]. C) Leukocytes of animal #408 (wildtype sow) spiked with leukocytes from #517 (ratio #408 : #517 = 100∶ 1). A Venus-positive leukocyte (+) is indicated. D) Corresponding brightfield illumination of C). Bar = 25 µm. (TIF) [file pone.0096673.s001.tif]
