# Supplementary material for: Assessment of Fetal Cell Chimerism in Transgenic Pig Lines Generated by Sleeping Beauty Transposition
Source: PLoS One. 2014 May 8;9(5):e96673. doi: 10.1371/journal.pone.0096673 (PMC4014516; doi:10.1371/journal.pone.0096673)
Supplement: Table S1 — Primer pairs used for RT-PCR. (DOCX) [file pone.0096673.s002.docx]

Supporting Information Table S1. Primer pairs used for RT-PCR

|  | Sequence | Annealing temperature | No. of cycles | | Amplification efficiency | | | Amplicon length |
| --- | --- | --- | --- | --- | --- | --- | --- | --- |
| CAGGS-Promoter  PAPOLA | 5´-GCAGCCACAGAAAAGAAACGA  5´- GCTCTGACTGACCGCGTTACT  5´- AGCCCCCAAGGAGACTGACT  5´- CTGACCTGCGCTGCAGTTC | 61  60 | | 45  45 | | 97 %  100% | 110 bp  101 bp | |
